# Subxiphoid Chest Tube Insertion for Extrapericardial Pneumomediastinum: A Simple and Effective Nonsurgical Treatment

**DOI:** 10.7759/cureus.84733

**Published:** 2025-05-24

**Authors:** Ananbabu Palaniappan, Farah Ghazali, Jazmine Mohd Ramzisham, Muhammad Ishamuddin Ismail, Mohd Ramzisham B Abdul Rahman

**Affiliations:** 1 Cardiothoracic Surgery, Universiti Kebangsaan Malaysia Medical Centre, Kuala Lumpur, MYS; 2 General Surgery, Universiti Kebangsaan Malaysia Medical Centre, Kuala Lumpur, MYS; 3 Medicine, Royal College of Surgeons in Ireland, Dublin, IRL

**Keywords:** cardiac tamponade, extrapericardial pneumomediastinum, macklin effect, minimally invasive technique, nonsurgical treatment, subxiphoid chest tube insertion, traumatic pneumomediastinum

## Abstract

Extrapericardial pneumomediastinum (EPM) is a rare condition characterized by the accumulation of air in the mediastinum outside the pericardial sac. It may present with symptoms such as dysphonia, shortness of breath, and neck or chest pain; however, it is often asymptomatic. In the absence of a history of trauma or foreign body aspiration, treatment is usually supportive. The condition is generally benign and self-limiting. While conservative management is often satisfactory, more invasive interventions may be required in severe cases. We report a case of EPM secondary to trauma that was successfully treated with subxiphoid chest tube placement.

## Introduction

Extrapericardial pneumomediastinum (EPM) is an uncommon condition characterized by the accumulation of air in the mediastinal space, but not within the pericardial sac [1]. It can arise from various causes, including trauma, infection, or spontaneous events [2]. While EPM is typically managed conservatively, severe cases may require invasive interventions [3]. The incidence of EPM among individuals aged 5-34 is approximately 1 in 25,000, with men comprising 76% of reported cases [1]. This case report presents subxiphoid chest tube insertion as a simple and effective nonsurgical treatment for EPM. It discusses the Macklin effect as a key component in understanding the condition's pathophysiology.

## Case presentation

**Figure 1:**
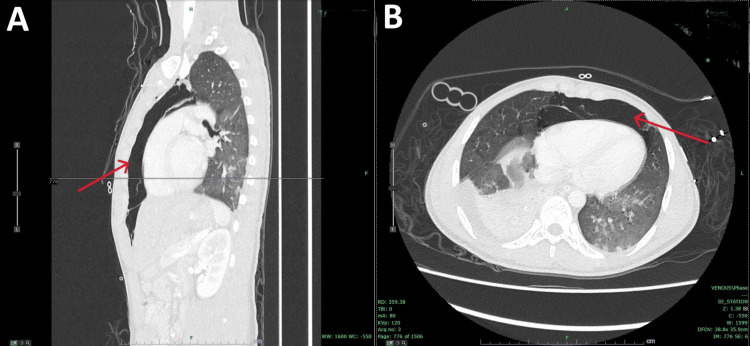
Computed tomography of the chest (A) Sagittal CT view showing pneumomediastinum (red arrow). (B) Axial CT view demonstrating pneumomediastinum (red arrow).

**Figure 2:**
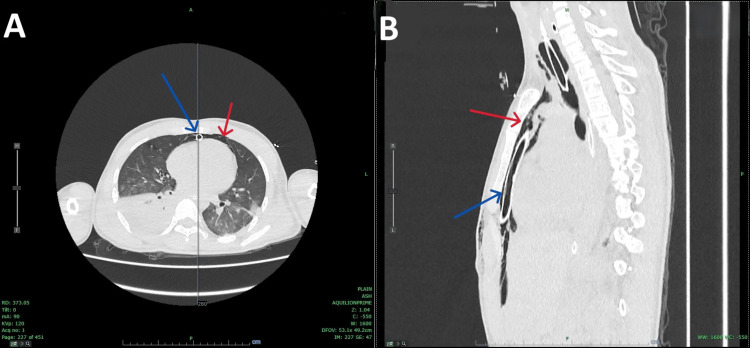
Computed tomography of the chest following insertion of the mediastinal drain (A) Axial CT view showing the mediastinal drain (blue arrow) and a significant reduction in pneumomediastinum (red arrow). (B) Sagittal CT view demonstrating the mediastinal drain (blue arrow) and markedly reduced pneumomediastinum (red arrow).

A 17-year-old male patient was admitted following a road traffic accident. He sustained an extradural hemorrhage, subarachnoid hemorrhage, and multiple facial fractures. A CT scan revealed EPM, without evidence of pneumothorax (Figures 1A, 1B). Urgent bronchoscopy was performed, but no trace of tracheobronchial injury was identified. Given the presence of bilateral lung contusions, the Macklin effect was suspected as the underlying mechanism. Bradycardia was noted and attributed to severe EPM, prompting the need for immediate intervention. Although echocardiography showed no pericardial effusion, CT imaging demonstrated worsening EPM with posterior displacement of the heart. Clinically, the patient developed progressive subcutaneous emphysema and signs of cardiac tamponade. Considering the risk of open surgical intervention, the decision was made to decompress the mediastinum. A straight 28 Fr chest tube was inserted retrosternally via a small subxiphoid incision, guided by CT imaging. A 14 G Venflon was used to mark the insertion site, minimizing the risk of injury to surrounding structures. Upon breaching the EPM, a large volume of air escaped through the chest drain. The tube was connected to a Sinapi Heimlich valve bottle. A repeat CT scan showed immediate and marked resolution of the EPM (Figures 2A, 2B), and the patient remained stable overnight. However, 24 hours later, the patient became hemodynamically unstable, exhibiting hypertension and bradycardia. Further investigation revealed brainstem herniation (“coning”) due to severe traumatic brain injury. In light of the poor prognosis, a decision was made to pursue conservative management. The patient passed away 24 hours later.

## Discussion

Our case was diagnosed as EPM secondary to the Macklin effect, a significant mechanism in the development of pneumomediastinum first proposed by Macklin in 1939 [4]. The Macklin effect explains how a sudden increase in intrathoracic pressure, due to events such as severe coughing, vomiting, or trauma, can lead to alveolar rupture. This occurs when the elevated pressure exceeds the structural integrity of the alveolar walls. Air then escapes into the perivascular and peribronchial interstitial spaces and tracks along the bronchovascular sheaths toward the mediastinum, resulting in pneumomediastinum. Due to underdiagnosis and the likelihood that many affected individuals do not seek medical attention, pneumomediastinum is believed by many experts to be more common than previously recognized. Furthermore, chest radiographs may not always reveal the condition, and associated symptoms are sometimes misdiagnosed as musculoskeletal pain or other benign causes. CT scan remains the most sensitive imaging modality for detecting pneumomediastinum and its complications [5].

In the presented case, EPM led to a tamponade effect, causing compression of the great vessels and impaired venous return [6]. Therefore, an emergency surgical intervention was needed. We utilized a subxiphoid chest tube insertion technique guided by CT, which proved to be a successful, minimally invasive, safe, and reproducible approach. Although subxiphoid chest tube placement was effective in this case, various other drainage techniques have been described for managing pneumomediastinum. Thoracoscopic mediastinotomy is a minimally invasive method that allows for mediastinal drainage under thoracoscopic guidance [7]. Another option is transverse cervicotomy with Penrose drain placement, which accesses the mediastinum through a cervical incision [8]. Classical mediastinal drainage involves dual incisions at the suprasternal notch and subxiphoid region for continuous suction. Additionally, the suprajugular mediastinal approach without drain placement has been employed to relieve tension while avoiding indwelling drains [9].

These techniques vary in complexity and invasiveness but share the common goal of safely managing EPM. Among them, subxiphoid chest tube insertion represents a less invasive alternative with promising outcomes.

## Conclusions

Subxiphoid chest tube insertion represents a successful and minimally invasive treatment option for EPM, as demonstrated in this case. The pathophysiological role of the Macklin effect in EPM emphasizes the importance of understanding the underlying mechanisms to guide appropriate therapeutic interventions. This case underscores the potential of subxiphoid drainage as a viable alternative to more invasive procedures and calls for further research to optimize EPM management strategies. Notably, the poor outcome in this patient was attributable to severe traumatic brain injury and was unrelated to the management of EPM.
